# Trend of hand, foot and mouth disease before, during, and after China’s COVID control policies in Zhejiang, China

**DOI:** 10.3389/fpubh.2024.1472944

**Published:** 2024-11-19

**Authors:** Zheyuan Ding, Qinbao Lu, Haocheng Wu, Chen Wu, Junfen Lin, Xinyi Wang, Tianying Fu, Ke Yang, Queping Song

**Affiliations:** ^1^Zhejiang Provincial Center for Disease Control and Prevention, Hangzhou, Zhejiang, China; ^2^Hangzhou Medical College, Hangzhou, Zhejiang, China

**Keywords:** COVID-19, hand, foot and mouth disease, interrupted time-series, non-pharmaceutical interventions, seasonal index

## Abstract

**Objective:**

To describe the trends in the incidence of hand, foot and mouth disease (HFMD) before, during, and after China’s Coronavirus Disease (COVID) control policies, and to interpret the influence on HFMD incidence at different control stages in Zhejiang Province.

**Methods:**

We collected data on HFMD cases in Zhejiang between 2014 and 2023. We compared the constituent ratios of cases at different COVID control stages by sex, age, child groups, and pathogens and weekly seasonal indices to observe seasonal variations in the incidence of HFMD. An interrupted time-series segmented regression analysis was applied to estimate the influence on HFMD incidence at different control stages. Stratified and sensitivity analyses were conducted to validate the findings.

**Results:**

A considerable proportion of cases occurred among children living separately. The proportions of children in kindergartens or nurseries and children aged 2–4 years were relatively low at the strict control stage compared to the other three stages. Enteroviruses other than enterovirus 71 and coxsackie virus A16 were the dominant HFMD pathogens, and the proportion showed an increasing trend. The usual spring–summer peak in HMFD incidence did not occur in 2020, and the periodicity of the biennial peak was disrupted for a year. The summer peak in 2023 was higher than that in the other years, and was delayed by 3 weeks. The trend changes in weekly HFMD cases during the strict control and regular control stages were − 15% (IRR: 0.85, 95% CI: 0.81–0.89) and 17% (IRR: 1.17, 95% CI: 1.12–1.23), respectively. However, the change was not statistically significant during the reopening stage (IRR: 1.41, 95% CI: 0.34–5.88). The expected number of cases increased by 1.12 times (95% CI: 243.17, 53.45%) during the reopening stage compared to what would have occurred if the zero-COVID policy had continued in 2023.

**Conclusion:**

Non-pharmaceutical interventions (NPIs) for COVID-19 control can mitigate HFMD. However, after the dynamic zero-COVID policy ended, the HFMD incidence returned to historical levels. Strict NPIs such as traffic restrictions and kindergarten closures cannot be sustained long-term. NPIs such as improving personal hygiene for routine prevention are highly recommended.

## Background

Hand, foot and mouth disease (HFMD) is a common infectious disease caused by a host of intestinal viruses, among which enterovirus 71 (EV71) and coxsackie virus A16 (CV-A16) are the most common (1). Its incidence is high in children younger than 5 years, especially in those aged 12–23 months (1, 2). HFMD has been a C-class notifiable infectious disease in China since 2008 (3), with morbidity and mortality rates among the highest for notifiable infectious diseases (4), representing a major disease burden. Zhejiang, a southeastern coastal province, has experienced one of the highest HFMD incidences in recent years (5). The number of cases increased rapidly between 2009 and 2016, ranking first among notifiable diseases (6).

The outbreak of severe acute respiratory syndrome coronavirus 2 (SARS-CoV-2), which causes Coronavirus Disease 2019 (COVID-19), began in early 2020 in China. Non-pharmaceutical interventions (NPIs) were immediately and widely implemented to prevent the transmission of the virus. Consequently, the incidence of HFMD decreased remarkably, and epidemic peaks were either absent or delayed in the first half of 2020 across mainland China (7), including Zhejiang (5, 8).

After 3 years of pandemic control measures, China ended its dynamic zero-COVID policy and implemented a reopening policy. From January 8 2023, COVID-19 was managed with measures against Class B infectious diseases, instead of Class A (9). Following the reopening, daily surveillance indicated a remarkable increase in HFMD incidence. This study aimed to describe the trend of HFMD incidence before, during, and after China’s COVID control policies and to interpret the influence of different COVID control stages on HFMD incidence in Zhejiang Province.

## Methods

### HFMD data

Data on HFMD cases in Zhejiang Province from 2014 to 2023 was acquired from the National Notifiable Infectious Disease Reporting Information System, part of the China Information System for Disease Control and Prevention. All cases included in the study were either clinically diagnosed or laboratory confirmed.

### COVID control stages

An emergency response was initiated on January 23 2020, in Zhejiang Province to control the COVID-19 outbreak. A series of stringent measures, such as regional lockdowns, traffic restrictions, suspending public gatherings and school closures, were implemented. With the gradual control of COVID-19 after 2–3 months, strict measures were removed. Kindergartens gradually reopened in May and June. Zhejiang Province entered into regular epidemic prevention and control period, and then dynamic zero-COVID policy was implemented until January 7 2023.

**Figure 1:**
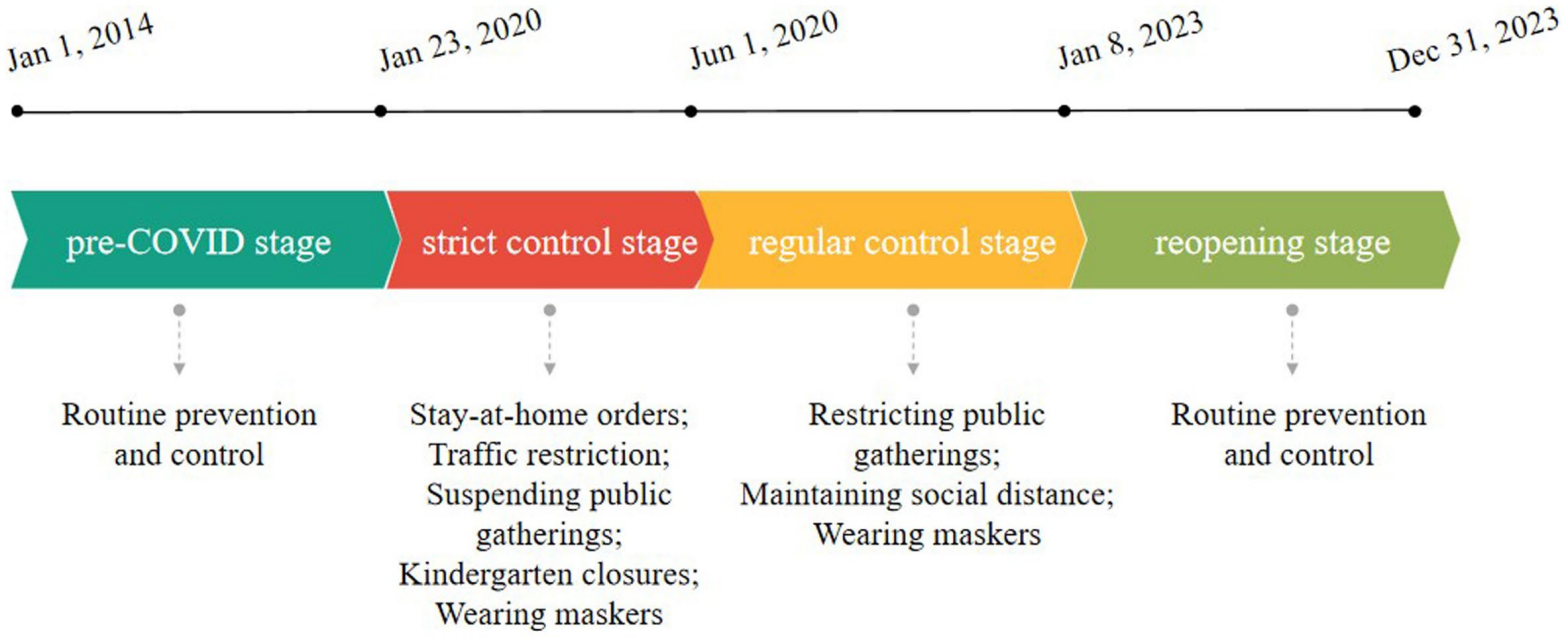
Timelines of COVID-19 and main non-pharmaceutical interventions in Zhejiang, 2014–2023.

Four stages were defined in this study (Figure 1): (1) pre-COVID stage: from January 1, 2014 to January 22, 2020; (2) Strict control stage: from January 23, 2020 to May 31, 2020; (3) Regular control stage: from June 1, 2020 to January 7, 2023; (4) Reopening stage: from January 8, 2023 to December 31, 2023.

### Statistical analysis

We calculated the number of HFMD cases across these four stages and compared the constituent ratios at different stages based on sex, age, child groups, and pathogens. The number of cases by pathogens has been recorded since 2016 when EV71 vaccination was launched (10).

Weekly seasonal indices (11) were calculated for four periods: 2014–2019 (pre-COVID stage), 2020 (strict to regular control stage), 2021–2022 (regular control stage), and 2023 (reopening stage). The index for a given week in a specific period was calculated by dividing the average case number for that week by the average weekly cases in the corresponding period. The indices according to sex, age, child groups and serotype were also calculated. Figures were drawn using WPS Office 2016.

An interrupted time-series segmented regression analysis was applied to estimate the influence of different COVID control stages on HFMD incidence. The equation is as follows:


$$Y_{t}\sim \text{quasiPossion}\left(\mu_{t}\right)$$

$$\begin{array}{l} \log\left(E\left(Y_{t}\right)\right)=\beta_{0}+\beta_{1}time_{t}+\beta_{2}\text{strictcontro}l_{t}\\ \;+\beta_{3}\text{time}\;\text{after}\;\text{strict}\;\text{contro}l_{t}+\beta_{4}\text{regularcontro}l_{t}\\ \;+\beta_{5}\text{time}\;\text{after}\;\text{regularcontro}l_{t}+\beta_{6}\text{reopenin}g_{t}\\ \;+\beta_{7}\text{time}\;\text{after}\;\text{reopenin}g_{t}+\beta_{8}\text{seasonality}\\ \;+\beta_{9}\text{holida}y_{t}+\log\left(\text{populatio}n_{t}\right)+\varepsilon_{t} \end{array}$$

where *Y_t_* is the number of HFMD cases in week *t*; *time_t_* is a continuous variable indicating time in weeks from 2014 to 2023; *strict control_t_*, *regular control_t_*, and *reopening_t_* are indicators for time *t* set to 0 before the intervention and to 1 after the intervention; *time after strict control_t_*, *time after regular control_t_*, and *time after reopening_t_* are continuous variables counting the number of weeks after the intervention; *holiday_t_* is a binary covariable of school holidays; Fourier term is added in the model to control seasonality; *population_t_* is the population in week t; *ε_t_* is the error term.

The level and trend changes in HFMD cases under different COVID control policies were expressed as incidence rate ratios (IRR) and 95% confidence intervals (CI). By setting the intervention terms and time after the intervention terms to zero, we estimated predicted cases in a counterfactual scenario. Two counterfactual scenarios were assumed: one where COVID-19 never occurred; and another where the reopening policy was not implemented. The relative change of HFMD cases during each stage was calculated as (observed cases—predicted cases)/predicted cases, expressed as a percentage change.

Durbin–Watson test was used to detect autocorrelation. Owing to the presence of autocorrelation, we used the Newey–West method (12, 13) to adjust standard errors. Stratified analyses were conducted based on sex, age, child groups and pathogens. Sensitivity analyses using 1-week lag and 2-week lags were conducted. Additionally, we conducted another sensitivity analysis by resetting March 23, 2020— when the emergency response was lowered to Level 3 in Zhejiang (8) —as a new boundary between the strict and regular control stage. All analyses were conducted using R software (version 4.1.2) with the packages “tsModel,” “splines,” “sandwich” and “lmtest.” A two-sided *p*-value <0.05 was considered statistically significant.

## Results

The proportion of HFMD cases was higher in men than women and did not vary considerably across the four stages. Children under 2 years of age accounted for 50.2% of HFMD cases in the strict control stage, a higher percentage than observed in the other three stages. In contrast, the proportion of children aged 2–4 years was higher in the other three stages than in the strict-control stage, accounting for approximately 50% of all cases. The proportion of cases in children over 5 years of age increased over time. Throughout all four stages, most cases of HFMD occurred in children living separately. At the strict control stage, the proportion of children living separately was relatively high, while that of children in kindergartens or nurseries was relatively low compared to the other three stages. Enteroviruses other than EV71 and CV-A16 were the dominant pathogens of HFMD, with their proportion showing an increasing trend, whereas the proportions of EV71 and CV-A16 decreased (Table 1).

**Table 1:** Numbers of hand, foot and mouth disease cases in different COVID control stages in Zhejiang, China.

	Pre-COVID stage	Strict control stage	Regular control stage	Reopening stage
Total (*n*)	956,747	1,215	242,565	148,011
Sex (*n*, %)				
Male	565,628 (59.1)	673 (55.4)	142,309 (58.7)	88,938 (60.1)
Female	391,119 (40.9)	542 (44.6)	100,256 (41.3)	59,073 (39.9)
Age (*n*, %)				
<2 years	364,583 (38.1)	610 (50.2)	72,693 (30.0)	45,490 (30.7)
2–4 years	469,075 (49.0)	412 (33.9)	125,842 (51.9)	67,857 (45.8)
≥5 years	123,089 (12.9)	193 (15.9)	44,030 (18.2)	34,664 (23.4)
Groups (*n*, %)				
Children living separately	635,545 (66.4)	906 (74.6)	130,199 (53.7)	81,736 (55.2)
Children in kindergartens or nurseries	279,148 (29.2)	199 (16.4)	93,213 (38.4)	48,769 (32.9)
Students	37,824 (4.0)	96 (7.9)	17,594 (7.3)	15,854 (10.7)
Others	4,230 (0.4)	14 (1.2)	1,559 (0.6)	1,652 (1.1)
Pathogens (*n*, %)^a^				
Subtotal (*n*)	23,165	41	9,431	4,525
EV71	3,588 (15.5)	10 (24.4)	514 (5.5)	315 (7.0)
CV-A16	4,954 (21.4)	6 (14.6)	1,472 (15.6)	497 (11.0)
Other enteroviruses	14,623 (63.1)	25 (61.0)	7,445 (78.9)	3,713 (82.1)

^aCounted since 2016, when EV71 vaccination was launched. EV71, enterovirus 71; CV-A16, coxsackie virus A16.^

**Figure 2:**
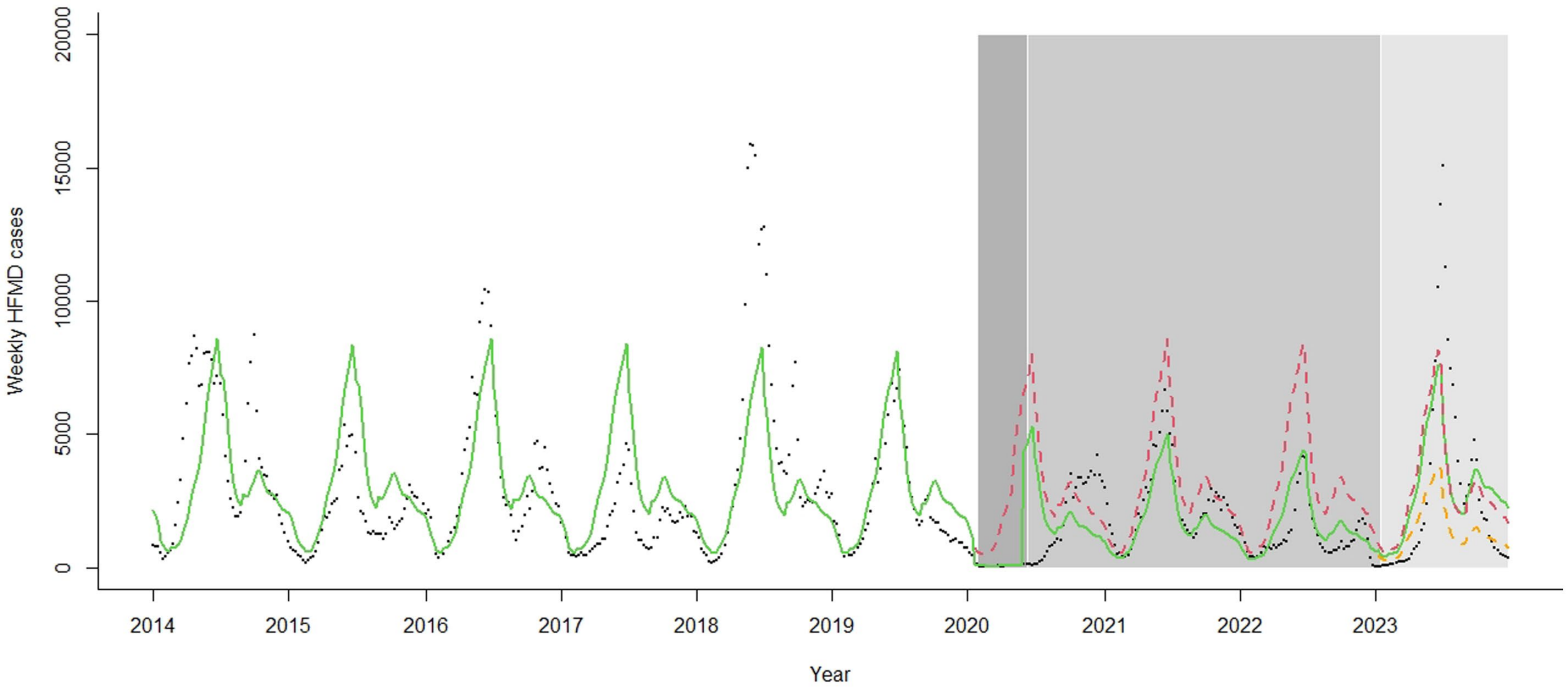
Trend in weekly hand, foot and mouth disease cases in Zhejiang, China, 2014–2023. The black dots represent observed cases. The green line represents the fitted value by the model. The red dashed line represents the estimated cases on counterfactual scenario that COVID-19 never occurred. The orange dashed line represents the estimated cases on counterfactual scenario where the reopening policy was not implemented. The white, dark grey, medium grey, and light grey zones represent the pre-COVID, strict control, regular control, and reopening stages, respectively. HFMD, hand, foot and mouth disease.

**Figure 3:**
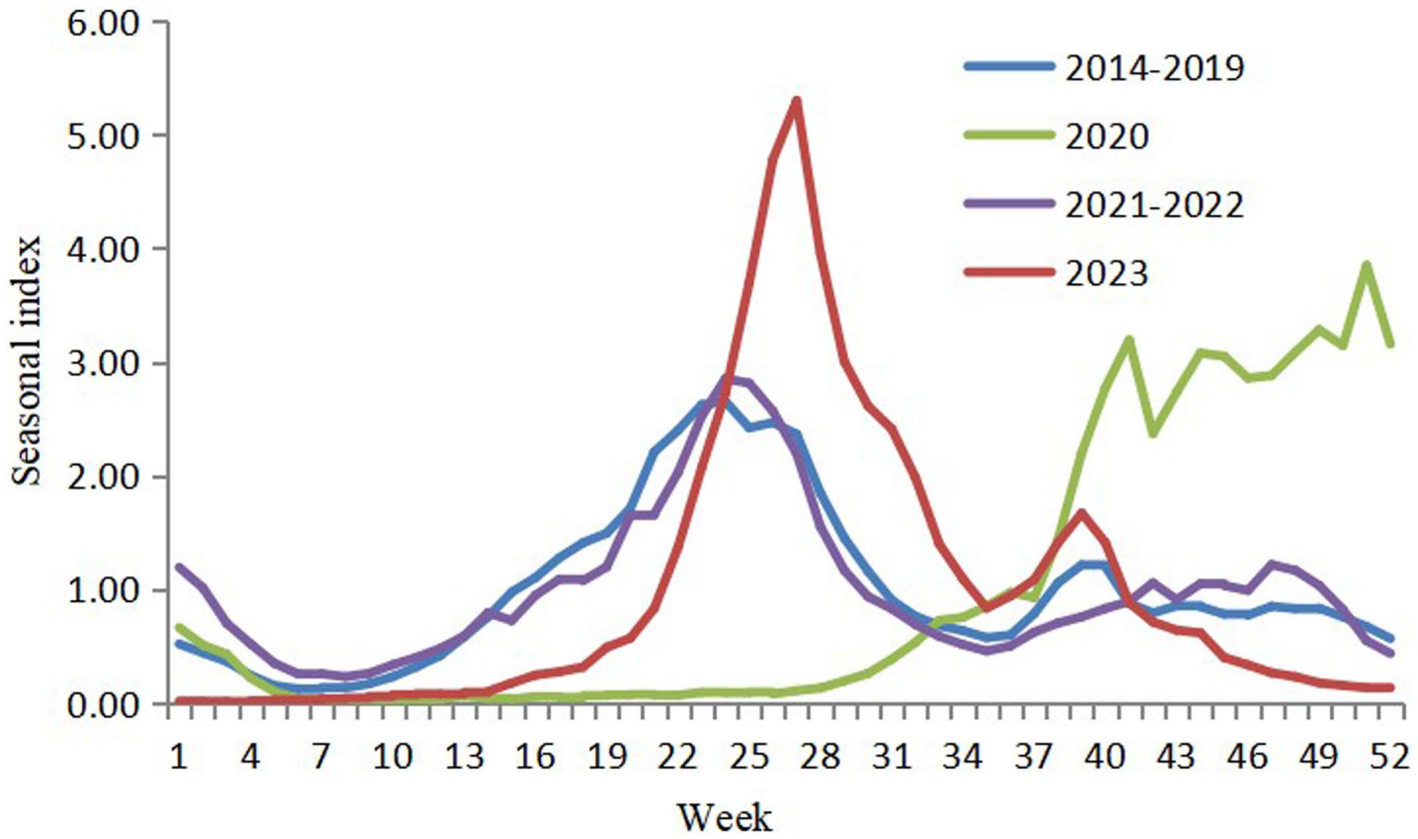
Weekly seasonal indices in hand, foot and mouth disease in Zhejiang, China, 2014–2023.

There were two peaks of HFMD cases every year in the pre-COVID stage: a higher peak from late spring to early summer and a lower peak in autumn. However, after the COVID-19 outbreak, only one peak was observed in 2020, occurring from autumn to early winter. The seasonal pattern in 2021–2022 returned to being similar to that in the pre-COVID stage. After the reopening, the incidence of HFMD was relatively low at the beginning of 2023. The first peak occurred in summer, approximately 3 weeks later than those observed in 2014–2019 and 2021–2022. Additionally, it showed a much higher peak than those in the other years. During the pre-COVID stage, more cases were reported in even-numbered years than in odd-numbered years. However, this alternating-year pattern has shifted since 2020, with more cases now occurring in odd-numbered years than in even-numbered years (Figures 2, 3).

The seasonal pattern did not vary considerably by sex, age, or child groups at pre-COVID stage. After the COVID-19 outbreak, the pattern remained similar between sexes, but differed across age and child groups. In 2020, the incidence peaked in early winter among children over 5 years old, children aged 2–4 years, children attending kindergartens or nurseries, and students, whereas only a smooth peak was observed among children under 2 years and children living separately. In 2023, the autumn peak was higher among children over 5 years and students, while the summer peak was lower in these groups. Among children under 2 years and children living separately, the autumn peak was not obvious. Autumn-winter peak of EV71 and summer peak of CV-A16 and other enteroviruses became obvious in 2023 compared to those in the pre-COVID stage (Supplementary Figures S1–S4).

Compared to the pre-COVID stage, the number of HFMD cases decreased by 81% (IRR: 0.19, 95% CI: 0.10–0.37) in the first week of the strict control stage, and the weekly cases decreased by a trend of 15% (IRR: 0.85, 95% CI: 0.81–0.89) under strict control measures. When the regular control stage began, the number of HFMD cases rebounded (IRR: 75.67, 95% CI: 23.25–246.27). Compared to the strict control stage, weekly cases increased by a trend of 17% (IRR: 1.17, 95% CI: 1.12–1.23) under regular control measures. After the zero-COVID policy was lifted, there was no significant immediate change in level (IRR: 1.41, 95% CI: 0.34–5.88) or trend (IRR: 1.02, 95% CI: 0.97–1.06) compared to the regular control stage. Stratified and sensitivity analyses showed similar results (Table 2 and Supplementary Tables S1, S2).

**Table 2:** Influence of different COVID control stages on hand, foot and mouth disease in Zhejiang, China.

	Level change after strict control	Trend change after strict control	Level change after regular control	Trend change after regular control	Level change after reopening	Trend change after reopening
	IRR(95% CI)	IRR(95% CI)	IRR(95% CI)	IRR(95% CI)	IRR(95% CI)	IRR(95% CI)
Overall	0.19 (0.10,0.37)***	0.85 (0.81,0.89)***	75.67 (23.25,246.27)***	1.17 (1.12,1.23)***	1.41 (0.34,5.88)	1.02 (0.97,1.06)
Sex						
Male	0.20 (0.10,0.37)***	0.84 (0.80,0.89)***	83.99 (26.28,268.40)***	1.18 (1.13,1.24)***	1.44 (0.34,6.02)	1.02 (0.97,1.06)
Female	0.18 (0.09,0.36)***	0.86 (0.81,0.90)***	66.39 (19.92,221.24)***	1.16 (1.10,1.22)***	1.36 (0.32,5.69)	1.02 (0.97,1.06)
Age						
<2 years	0.23 (0.12,0.47)***	0.86 (0.82,0.91)***	42.18 (13.26,134.20)***	1.15 (1.10,1.21)***	2.01 (0.45,9.02)	1.01 (0.97,1.06)
2–4 years	0.16 (0.08,0.32)***	0.83 (0.79,0.88)***	155.37 (41.96,575.28)***	1.20 (1.13,1.27)***	1.38 (0.33,5.80)	1.01 (0.97,1.06)
≥5 years	0.22 (0.12,0.39)***	0.85 (0.81,0.89)***	57.16 (18.84,173.49)***	1.18 (1.13,1.24)***	0.84 (0.21,3.42)	1.03 (0.98,1.07)
Groups						
Children living separately	0.21 (0.11,0.41)***	0.86 (0.81,0.90)***	56.86 (16.97,190.52)***	1.16 (1.11,1.23)***	1.77 (0.39,8.00)	1.01 (0.97,1.06)
Children in kindergartens or nurseries	0.14 (0.06,0.31)***	0.84 (0.78,0.89)***	176.76 (49.43,632.08)***	1.19 (1.12,1.27)***	1.15 (0.28,4.66)	1.01 (0.97,1.06)
Students	0.29 (0.16,0.52)***	0.86 (0.82,0.90)***	33.52 (13.26,84.76)***	1.16 (1.11,1.22)***	1.10 (0.30,4.09)	1.02 (0.98,1.06)
Pathogens						
EV71	0.40 (0.17,0.95)*	0.84 (0.77,0.92)***	42.69 (7.79,233.84)***	1.19 (1.09,1.29)***	0.66 (0.17,2.60)	1.05 (1.02,1.09)**
CV-A16	0.06 (0.03,0.12)***	0.84 (0.80,0.89)***	118.61 (49.54,283.97)***	1.19 (1.13,1.25)***	0.30 (0.11,0.78)*	1.02 (1.00,1.06)
Other enteroviruses	0.09 (0.05,0.16)***	0.88 (0.86,0.91)***	107.68 (43.92,263.99)***	1.12 (1.08,1.16)***	2.73 (0.80,9.34)	1.00 (0.97,1.04)

IRR, incidence rate ratio; CI, confidence intervals; EV71, enterovirus 71. CV-A16, coxsackie virus A16. *p < 0.05, **p < 0.01, ***p < 0.001.

During the strict control stage, the number of HFMD cases reduced by 96.82% (95% CI: −95.63% – –97.51%) compared to the expected number without the implementation of the strict policies. The results were similar across the stratified analyses. In the regular control stage, the reduction was 42.49% (95% CI: −23.41% – –53.95%) compared to a scenario where COVID control policies were never implemented, with similar results between sexes. However, the reduction was lower in children aged 2–4 years than in other age groups. The reduction in children in kindergartens or nurseries was less than that in children living separately and students. The protective effect of COVID control policies was greater among HFMD cases caused by CV-A16 than among those caused by EV71 or other enteroviruses, with a reduction of 60.02% (95% CI: −47.48% – –67.73%). Regardless of the COVID-19 outbreak, the total number of HFMD cases did not change considerably in the reopening stage (relative change: –1.98%, 95% CI: 40.60% – –24.77%). However, it increased by 1.12 times (95% CI: 243.17–53.45%) compared to what it would have been if the zero-COVID policy had continued in 2023. Stratified analyses showed similar results except for HFMD cases caused by CV-A16, which showed a decrease (relative change: –45.62%, 95% CI: −12.96% – –60.43%) (Table 3).

**Table 3:** Relative changes of hand, foot and mouth disease cases during different COVID control stages in Zhejiang, China.

	Strict control stage^a^			Regular control stage^a^			Reopening stage^a^			Reopening stage^b^		
	Observed cases (*n*)	Predicted cases (n, 95% CI)	Relative change (%,95% CI)	Observed cases (n)	Predicted cases (*n*, 95% CI)	Relative change (%,95% CI)	Observed cases (n)	Predicted cases (*n*, 95% CI)	Relative change (%, 95% CI)	Observed cases (*n*)	Predicted cases (*n*, 95% CI)	Relative change (%, 95% CI)
Overall	1215	38250(27781,48718)	–96.82 (–95.63, –97.51)	242565	421748 (316712,526784)	–42.49 (–23.41,–53.95)	148011	151002 (105270,196735)	–1.98 (40.60,–24.77)	148011	69794 (43130,96457)	112.07 (243.17,53.45)
Sex												
Male	673	22086 (16038,28133)	–96.95 (–95.80,–97.61)	142309	245428 (184228,306628)	–42.02 (–22.75,–53.59)	88938	87244 (60774,113714)	1.94 (46.34,–21.79)	88938	41357 (25517,57197)	115.05 (248.54,55.49)
Female	542	16179 (11747,20612)	–96.65 (–95.39,–97.37)	100256	176386 (132468,220305)	–43.16 (–24.32,–54.49)	59073	63811 (44505,83117)	–7.43 (32.73,–28.93)	59073	28438 (17597,39280)	107.73 (235.70,50.39)
Age												
<2 years	610	13225 (9411,17039)	–95.39 (–93.52,–96.42)	72693	156366 (116670,196063)	–53.51 (–37.69,–62.92)	45490	54442 (37604,71280)	–16.44 (20.97,–36.18)	45490	16249 (9106,23393)	179.96 (399.56,94.46)
2–4 years	412	18761 (13617,23904)	–97.80 (–96.97,–98.28)	125842	192388 (144144,240632)	–34.59 (–12.70,–47.70)	67857	67734 (47118,88350)	0.18 (44.02,–23.20)	67857	34792 (21708,47875)	95.04 (212.59,41.74)
≥5 years	193	6157 (4454,7861)	–96.87 (–95.67,–97.54)	44030	76480 (56172,96787)	–42.43 (–21.62,–54.51)	34664	31530 (21304,41756)	9.94 (62.71,–16.98)	34664	21008 (13641,28375)	65.00 (154.12,22.16)
Groups												
Children living separately	906	23517 (16927,30214)	–96.15 (–94.65,–97.00)	130199	260757 (195619,325894)	–50.07 (–33.44,–60.05)	81736	89775 (62473,117077)	–8.95 (30.83,–30.19)	81736	32216 (18638,45794)	153.71 (338.54,78.49)
Children in kindergartens or nurseries	199	11954 (8642,15265)	–98.34 (–97.70,–98.70)	93213	130369 (95812,164926)	–28.50 (–2.71,–43.48)	48769	49113 (33321,64905)	–0.70 (46.36,–24.86)	48769	29814 (18939,40688)	63.58 (157.51,19.86)
Students	96	1764 (1266,2262)	–94.56 (–92.42,–95.76)	17594	31956 (23503,40409)	–44.94 (–25.14,–56.46)	15854	14467 (9748,19187)	9.59 (62.64,–17.37)	15854	7908 (5337,10478)	100.48 (197.06,51.31)
Pathogens												
EV71	10	150 (107,192)	–93.33 (–90.65,–94.79)	514	741 (552,930)	–30.63 (–6.88,–44.73)	315	170 (118,223)	85.29 (166.95,41.26)	315	123 (52,195)	156.10 (505.77,61.54)
CV-A16	6	555 (407,704)	–98.92 (–98.53,–99.15)	1472	3682 (2803,4561)	–60.02 (–47.48,–67.73)	497	1507 (1080,1935)	–67.02 (–53.98,–74.32)	497	914 (571,1256)	–45.62 (–12.96,–60.43)
Other enteroviruses	25	929 (702,1155)	–97.31 (–96.44,–97.84)	7445	13814 (10838,16791)	–46.11 (–31.31,–55.66)	3713	5758 (4244,7273)	–35.52 (–12.51,–48.95)	3713	1217 (832,1603)	205.09 (346.27,131.63)

^aA scenario that COVID-19 never occurred. bA scenario that reopening policy was not implemented. CI, confidence intervals; EV71, enterovirus 71; CV-A16, coxsackie virus A16.^

## Discussion

Our study revealed that the incidence of HFMD decreased considerably during the COVID control policies in Zhejiang from 2020 to 2022. The usual summer peak in 2020 disappeared, and the alternate-year pattern shifted after 2020. Upon reopening, the incidence increased by 1.12 times compared to what it would have been if the zero-COVID policy had continued in 2023.

COVID control policies and NPIs produced a marked effect not only on controlling COVID-19 but also on other notifiable infectious diseases, like influenza, mumps, pertussis, scarlet fever, infectious diarrhea (14–17), and HFMD (7, 14, 18, 19). Similar results were observed in our study. NPIs, such as traffic restrictions and kindergarten closures, considerably reduced interactions among people, especially young children, who are the most susceptible to HFMD (1, 2). The decline in the levels and trends of HFMD incidence under strict control policies was evident. The number of HFMD cases during the strict-control stage was expected to decrease by 97%. The typical spring–summer peak of HFMD was absent. When strict NPIs were replaced by regular control measures, the HFMD incidence rebounded, and semiannual peaks reappeared. This was mainly due to the reopening of kindergartens and schools (8, 14). Nevertheless, the observed number of cases during the regular control stage was less than 60% of the expected number in the counterfactual scenario. HFMD can be transmitted via fecal-oral, oral-oral and respiratory routes (20), with fecal-oral transmission believed to be the principal route (21). Thus, the effect of routine prevention and control measures for COVID-19 (such as wearing masks) in the regular control stage was limited in preventing the transmission of HFMD. However, the COVID prevention and control measures improved health consciousness in parents and teachers, helping children maintain good personal health habits. Strengthened morning checks in kindergartens and schools also helped to reduce the transmission of infectious diseases among children.

Upon reopening, a trough in HFMD incidence was first observed followed by a summer peak. Owing to COVID control policies, a large population in China was never infected with SARS-CoV-2 and had very low pre-existing immunity by the end of 2022. When the zero-COVID policy ended, the Omicron variant spread rapidly, leading to a sudden surge in COVID-19 cases (22, 23). Many uninfected people chose to stay home to avoid infection, while many of infected stayed home to recover. Schools and kindergartens brought forward their winter holidays to mid-to-late December 2022, leading to a sharp decline in the transmission of HFMD and other infectious diseases. By February 2023, the COVID-19 epidemic had subsided (24), and society recovered. Schools and kindergartens gradually opened, and the number of HFMD cases rebounded. The incidence returned nearly to pre-COVID levels in Zhejiang, and the summer peak in 2023 came late and reached high level, especially among children under 5 years. A nationwide study conducted in China revealed similar results (25). The peak in HFMD incidence was delayed compared to the trends in previous years, and reached a higher epidemic level by the 26^th^ week. It appeared that the long-term effect of NPIs on HFMD did not persist after the reopening. The reasons might be as follows. First, the low epidemic of HFMD in the COVID-19 pandemic period likely resulted in a relatively low immune level for enteroviruses in children, leading to increased susceptibility to HFMD in the reopening stage (26). Second, after the reopening, population movements and social activities increased remarkably, facilitating the disease transmission. Third, after the 3-year COVID control period, people were no longer required to adhere to measures such as mask wearing, health code checking, nucleic acid testing, and travel restriction, potentially leading to a decline in personal health consciousness and health behaviors.

Schools and kindergartens are important locations for HFMD transmission. The timing of the autumn-winter peak in HFMD cases was coincided with the school semester in school and kindergarten children. Additionally, we found a decrease in the number of cases during the strict control stage when kindergartens and schools closed and a sharp early winter peak in 2020 when kindergartens and schools reopened, particularly among children at kindergartens or nurseries. These results prove that kindergarten closure is an effective measure to interrupt the transmission of HFMD, consistent with findings from other studies (7, 18). The constituent ratio of children living separately was always the highest at each stage, indicating community transmission was another important route of infection. School and kindergarten closures can only protect preschools and school children and cannot be a permanent measure in the long term. Moreover, community transmission still persists when children are out of school or kindergarten (2). Helping children establish good hygiene habits, such as hand washing, which is an effective measure against community-acquired HFMD (27), is easier to carry out than kindergarten closure and should be highly recommended.

HFMD incidence showed obvious periodicity and seasonality. It may be complicated by interference between the causative enterovirus serotypes and associated with climate factors like precipitations, sunshine, temperature, and air pressure (1, 28). The biennial peak of HFMD may be related to the peak incidence of pathogens: before 2017, the main contributors were EV71 and CV-A16, but since 2018, they have been gradually replaced by other enteroviruses (5). However, the drivers behind these patterns are not fully understood. The shift in the alternate-year pattern observed in our study indicated that the COVID-19 epidemic and NPIs in 2020 had a marked effect on controlling HFMD, delaying its periodicity by 1 year in Zhejiang.

Surveillance data have shown that the proportion of EV71 has decreased in China in recent years, while the prevalence of other enteroviruses, such as CV-A6 and CV-A10, has been rapidly increasing (5, 29). We observed a similar trend in our study. Three EV71 vaccines have been launched in China since 2016 (10), and many studies have demonstrated that these vaccines are vital in reducing EV71-related incidence and case-severity rates (5, 10, 30, 31). Following the widespread deployment of EV71 vaccines, EV71 prevalence decreased gradually from 2009 to 2016, likely due to the lower viral diversity of EV71 compared to CV-A16, CV-A6 and CV-A10, which reduces its fitness and transmission (29). These studies explain the decrease in EV71 prevalence. The proportion of cases among children over 5 years has increased in recent years, which may be partly due to the greater benefit of the EV71 vaccines in children under 5 years compared to those over 5 years (32). We also observed that the reduction in CV-A16 cases was highest at the regular control stage, which is consistent with findings from another study in Guangzhou, where researchers suspected that NPIs might have a stronger effect on CV-A16 than on other serotypes (18). However, in our study, the reduction in the number of CV-A16 cases during the reopening stage was also significant, indicating that factors other than NPIs may also contribute to the reduction in CV-A16 cases. Further research is needed to identify these potential drivers.

This study had some limitations. First, the HFMD data were acquired from a passive surveillance system and inevitably had a bias. For example, in the strict control stage, because of traffic restrictions, patients with mild symptoms may not seek medical attention; therefore, the number of HFMD cases may have been underestimated. Second, the boundary between the strict and regular control stages was difficult to define. Considering the characteristics of HFMD transmission, kindergarten reopening was set as the boundary instead of the change in emergency response level (March 23 2020). The recomputed results in the sensitivity analyses showed a smaller level change and a larger trend change at the strict-control stage, but the results remain consistent with the primary analysis. Third, the model did not account for possible confounding factors such as meteorological factors (33) and vaccination rates, restricting further in-depth analyses. Lastly, the serotyping rate was low, and data on serotype other than EV71 and CV-A16 were lacking, limiting further analysis of serotypes. Surveillance of other enteroviruses, such as CV-A6, should be included in the future.

In conclusion, our study demonstrated that after the zero-COVID policy ended in 2023, the incidence of HFMD increased considerably, returning to the level before the COVID-19 epidemic and showing a later and higher summer peak. Health workers should continuously monitor HFMD and maintain sharp vigilance regarding rebound after a period of low prevalence. They should also pay close attention to the influence of any public health policy termination, such as the cancellation of class suspensions, on HFMD, especially in young children. Strict NPIs, such as traffic restrictions and kindergarten closures, are efficient in controlling HFMD transmission but are not sustainable in the long term. Instead, NPIs such as strengthening morning checks in kindergartens and schools, educations and supervision of children’s personal hygiene in routine prevention, and HFMD vaccination, are recommended. With enteroviruses other than EV71 and CV-A16 becoming the dominant serotypes, virus typing for strains like CV-A6 and CV-A10 should be included in routine surveillance. Additionally, future studies should conduct more in-depth studies on serotype changes in HFMD and investigate potential causes.

## Data Availability

Data are available from the authors upon reasonable request and with permission of Provincial Center for Disease Control and Prevention. Requests to access the datasets should be directed to Zheyuan Ding, zhyding@cdc.zj.cn.

## Supplementary material

SUPPLEMENTARY FIGURE 1 Weekly seasonal indices in hand, foot and mouth disease by sexes in Zhejiang, China, 2014–2023.

SUPPLEMENTARY FIGURE 2 Weekly seasonal indices in hand, foot and mouth disease by age groups in Zhejiang, China, 2014–2023.

SUPPLEMENTARY FIGURE 3 Weekly seasonal indices in hand, foot and mouth disease by child groups in Zhejiang, China, 2014–2023.

SUPPLEMENTARY FIGURE 4 Weekly seasonal indices in hand, foot and mouth disease by serotypes in Zhejiang, China, 2016–2023. EV71: enterovirus 71. CV-A16: coxsackie virus A16.
