# Supplementary figures and images for: Trend of hand, foot and mouth disease before, during, and after China’s COVID control policies in Zhejiang, China
Source: Front Public Health. 2024 Nov 19;12:1472944. doi: 10.3389/fpubh.2024.1472944 (PMC11611829; doi:10.3389/fpubh.2024.1472944)

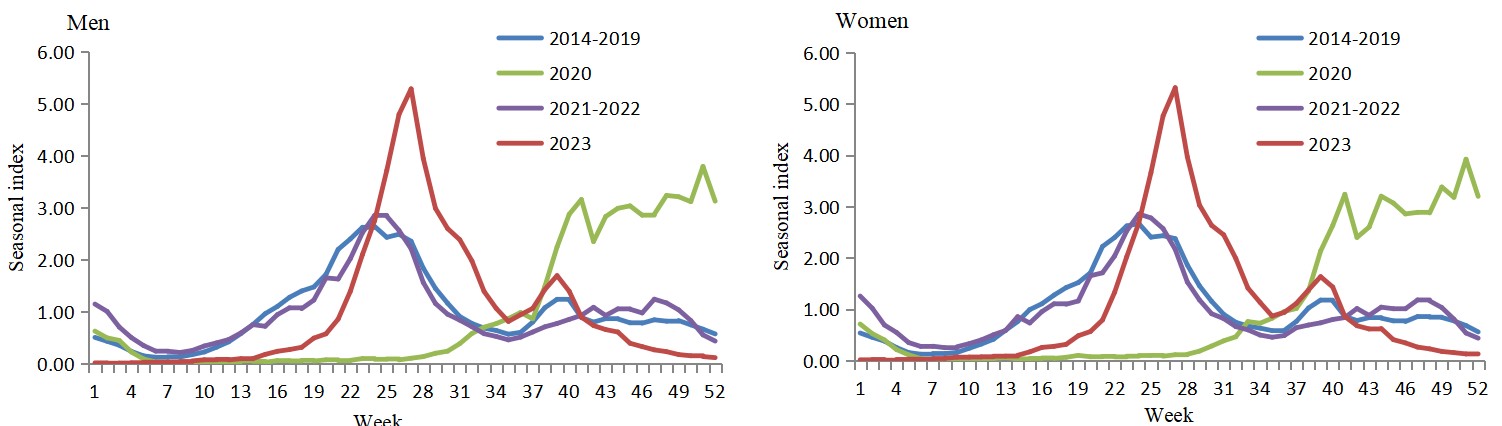

Supplement: SUPPLEMENTARY FIGURE 1 — Weekly seasonal indices in hand, foot and mouth disease by sexes in Zhejiang, China, 2014–2023. [file Image_1.JPEG]

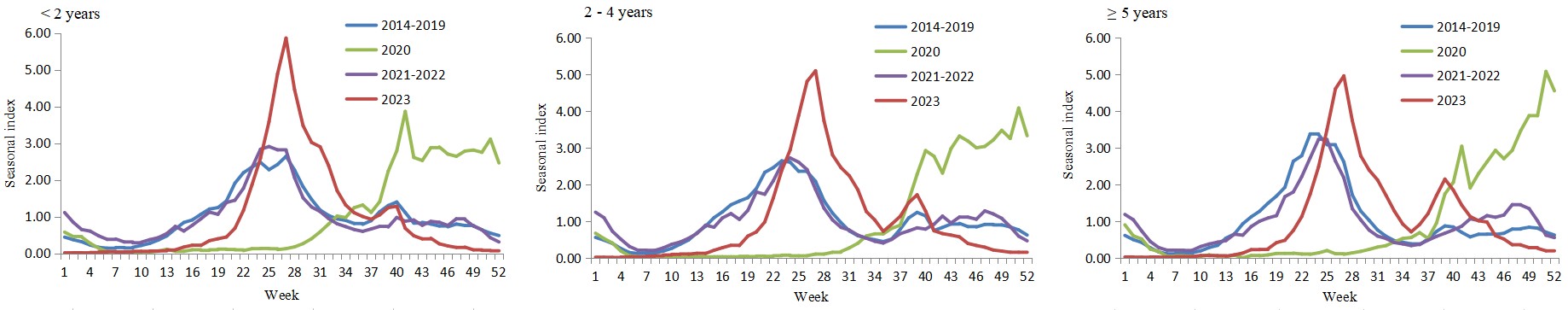

Supplement: SUPPLEMENTARY FIGURE 2 — Weekly seasonal indices in hand, foot and mouth disease by age groups in Zhejiang, China, 2014–2023. [file Image_2.JPEG]

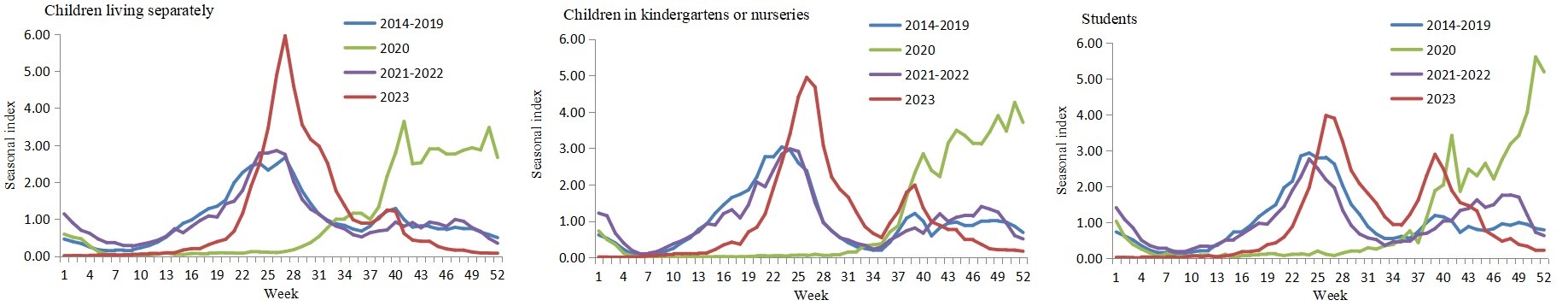

Supplement: SUPPLEMENTARY FIGURE 3 — Weekly seasonal indices in hand, foot and mouth disease by child groups in Zhejiang, China, 2014–2023. [file Image_3.jpeg]

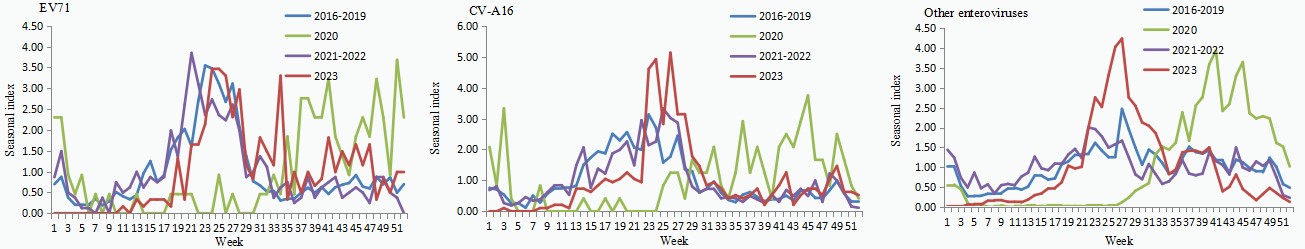

Supplement: SUPPLEMENTARY FIGURE 4 — Weekly seasonal indices in hand, foot and mouth disease by serotypes in Zhejiang, China, 2016–2023. EV71: enterovirus 71. CV-A16: coxsackie virus A16. [file Image_4.JPEG]
