# Supplementary material for: Trend of hand, foot and mouth disease before, during, and after China’s COVID control policies in Zhejiang, China
Source: Front Public Health. 2024 Nov 19;12:1472944. doi: 10.3389/fpubh.2024.1472944 (PMC11611829; doi:10.3389/fpubh.2024.1472944)
Supplement: Supplementary file 5 [file Table_1.DOCX]

| Supplemental Table 1 Influence of different COVID control stages on hand, foot and mouth disease with lag effects in Zhejiang, China | | | | | | |
| --- | --- | --- | --- | --- | --- | --- |
|  | Level change after strict control | Trend change after strict control | Level change after regular control | Trend change after regular control | Level change after reopening | Trend change after reopening |
|  | IRR（95%CI） | IRR（95%CI） | IRR（95%CI） | IRR（95%CI） | IRR（95%CI） | IRR（95%CI） |
| Overall | 0.19(0.10,0.37)^***^ | 0.85(0.81,0.89)^***^ | 75.67(23.25,246.27)^***^ | 1.17(1.12,1.23)^***^ | 1.41(0.34,5.88) | 1.02(0.97,1.06) |
| Overall (1-week lag) | 0.16(0.07,0.35)^***^ | 0.91(0.85,0.97)^**^ | 44.35(19.38,101.48)^***^ | 1.10(1.03,1.17)^**^ | 1.11(0.46,2.68) | 1.01(0.98,1.04) |
| Overall (2-week lags) | 0.18(0.07,0.44)^***^ | 0.89(0.83,0.96)^**^ | 56.11(24.73,127.32)^***^ | 1.12(1.04,1.20)^**^ | 1.37(0.52,3.57) | 1.01(0.98,1.04) |
| Sex |  |  |  |  |  |  |
| Male | 0.20(0.10,0.37)^***^ | 0.84(0.80,0.89)^***^ | 83.99(26.28,268.40)^***^ | 1.18(1.13,1.24)^***^ | 1.44(0.34,6.02) | 1.02(0.97,1.06) |
| Male (1-week lag) | 0.16(0.07,0.35)^***^ | 0.90(0.85,0.96)^**^ | 49.95(22.31,111.86)^***^ | 1.11(1.04,1.18)^**^ | 1.11(0.46,2.66) | 1.01(0.98,1.04) |
| Male (2-week lags) | 0.18(0.07,0.44)^***^ | 0.88(0.82,0.95)^***^ | 63.10(28.35,140.45)^***^ | 1.13(1.05,1.21)^***^ | 1.37(0.53,3.57) | 1.01(0.98,1.04) |
| Female | 0.18(0.09,0.36)^***^ | 0.86(0.81,0.90)^***^ | 66.39(19.92,221.24)^***^ | 1.16(1.10,1.22)^***^ | 1.36(0.32,5.69) | 1.02(0.97,1.06) |
| Female (1-week lag) | 0.16(0.07,0.35)^***^ | 0.92(0.86,0.98)^**^ | 38.19(15.81,92.27)^***^ | 1.09(1.02,1.16)^*^ | 1.12(0.45,2.79) | 1.01(0.98,1.04) |
| Female (2-week lags) | 0.17(0.07,0.45)^***^ | 0.90(0.83,0.97)^**^ | 48.42(21.53,108.91)^***^ | 1.11(1.03,1.20)^**^ | 1.36(0.56,3.33) | 1.01(0.98,1.03) |
| Age |  |  |  |  |  |  |
| <2 years | 0.23(0.12,0.47)^***^ | 0.86(0.82,0.91)^***^ | 42.18(13.26,134.20)^***^ | 1.15(1.10,1.21)^***^ | 2.01(0.45,9.02) | 1.01(0.97,1.06) |
| <2 years (1-week lag) | 0.22(0.11,0.42)^***^ | 0.91(0.87,0.96)^***^ | 29.73(12.91,68.50)^***^ | 1.09(1.04,1.15)^***^ | 1.66(0.52,5.24) | 1.01(0.97,1.05) |
| <2 years (2-week lags) | 0.23(0.10,0.56)^**^ | 0.90(0.84,0.96)^**^ | 37.09(16.93,81.29)^***^ | 1.11(1.04,1.19)^**^ | 1.91(0.62,5.86) | 1.01(0.98,1.04) |
| 2-4 years | 0.16(0.08,0.32)^***^ | 0.83(0.79,0.88)^***^ | 155.37(41.96,575.28)^***^ | 1.20(1.13,1.27)^***^ | 1.38(0.33,5.80) | 1.01(0.97,1.06) |
| 2-4 years (1-week lag) | 0.12(0.05,0.30)^***^ | 0.90(0.82,0.97)^**^ | 77.10(27.57,215.56)^***^ | 1.11(1.03,1.21)^*^ | 1.14(0.49,2.64) | 1.01(0.98,1.04) |
| 2-4 years (2-week lags) | 0.13(0.05,0.39)^***^ | 0.88(0.80,0.96)^**^ | 99.94(38.66,258.36)^***^ | 1.14(1.04,1.24)^**^ | 1.39(0.63,3.03) | 1.01(0.98,1.03) |
| ≥5 years | 0.22(0.12,0.39)^***^ | 0.85(0.81,0.89)^***^ | 57.16(18.84,173.49)^***^ | 1.18(1.13,1.24)^***^ | 0.84(0.21,3.42) | 1.03(0.98,1.07) |
| ≥5 years (1-week lag) | 0.18(0.10,0.33)^***^ | 0.90(0.85,0.94)^***^ | 41.03(16.63,101.25)^***^ | 1.12(1.06,1.17)^***^ | 0.56(0.27,1.16) | 1.02(1.00,1.05) |
| ≥5 years (2-week lags) | 0.23(0.12,0.45)^***^ | 0.87(0.83,0.92)^***^ | 48.59(19.33,122.16)^***^ | 1.14(1.09,1.20)^***^ | 0.82(0.33,2.04) | 1.01(0.98,1.04) |
| Groups |  |  |  |  |  |  |
| Children living separately | 0.21(0.11,0.41)^***^ | 0.86(0.81,0.90)^***^ | 56.86(16.97,190.52)^***^ | 1.16(1.11,1.23)^***^ | 1.77(0.39,8.00) | 1.01(0.97,1.06) |
| Children living separately (1-week lag) | 0.19(0.10,0.38)^***^ | 0.91(0.86,0.96)^***^ | 37.46(15.28,91.83)^***^ | 1.10(1.04,1.16)^***^ | 1.43(0.46,4.42) | 1.01(0.97,1.05) |
| Children living separately (2-week lags) | 0.21(0.08,0.50)^***^ | 0.89(0.83,0.95)^**^ | 47.96(20.43,112.57)^***^ | 1.12(1.04,1.20)^**^ | 1.70(0.55,5.19) | 1.01(0.97,1.04) |
| Children in kindergartens or nurseries | 0.14(0.06,0.31)^***^ | 0.84(0.78,0.89)^***^ | 176.76(49.43,632.08)^***^ | 1.19(1.12,1.27)^***^ | 1.15(0.28,4.66) | 1.01(0.97,1.06) |
| Children in kindergartens or nurseries (1-week lag) | 0.09(0.03,0.29)^***^ | 0.91(0.82,1.00) | 83.94(27.19,259.09)^***^ | 1.10(1.00,1.22) | 1.04(0.48,2.26) | 1.01(0.98,1.03) |
| Children in kindergartens or nurseries (2-week lags) | 0.11(0.03,0.37)^***^ | 0.89(0.80,0.98)^*^ | 104.19(37.35,290.65)^***^ | 1.13(1.02,1.24)^*^ | 1.26(0.54,2.93) | 1.00(0.98,1.03) |
| Students | 0.29(0.16,0.52)^***^ | 0.86(0.82,0.90)^***^ | 33.52(13.26,84.76)^***^ | 1.16(1.11,1.22)^***^ | 1.10(0.30,4.09) | 1.02(0.98,1.06) |
| Students (1-week lag) | 0.25(0.16,0.39)^***^ | 0.90(0.87,0.92)^***^ | 26.98(13.90,52.38)^***^ | 1.12(1.08,1.15)^***^ | 0.54(0.28,1.04) | 1.02(1.00,1.03) |
| Students (2-week lags) | 0.34(0.20,0.59)^***^ | 0.87(0.85,0.90)^***^ | 30.78(15.54,60.97)^***^ | 1.14(1.11,1.18)^***^ | 0.90(0.42,1.94) | 1.00(0.98,1.03) |
| Pathogens |  |  |  |  |  |  |
| EV71 | 0.40(0.17,0.95)^*^ | 0.84(0.77,0.92)^***^ | 42.69(7.79,233.84)^***^ | 1.19(1.09,1.29)^***^ | 0.66(0.17,2.60) | 1.05(1.02,1.09)^**^ |
| EV71 (1-week lag) | 0.20(0.07,0.54)^**^ | 0.89(0.80,0.98)^*^ | 32.69(6.11,175.01)^***^ | 1.12(1.02,1.24)^*^ | 0.93(0.25,3.47) | 1.04(1.01,1.07) |
| EV71 (2-week lags) | 0.22(0.07,0.64)^**^ | 0.87(0.78,0.98)^*^ | 39.01(6.65,228.95)^***^ | 1.14(1.02,1.27)^*^ | 0.89(0.23,3.44) | 1.04(1.01,1.08) |
| CV-A16 | 0.06(0.03,0.12)^***^ | 0.84(0.80,0.89)^***^ | 118.61(49.54,283.97)^***^ | 1.19(1.13,1.25)^***^ | 0.30(0.11,0.78)^*^ | 1.02(1.00,1.06) |
| CV-A16 (1-week lag) | 0.07(0.04,0.12)^***^ | 0.89(0.84,0.93)^***^ | 69.56(29.65,163.19)^***^ | 1.13(1.08,1.19)^***^ | 0.43(0.17,1.12) | 1.02(0.99,1.05) |
| CV-A16 (2-week lags) | 0.06(0.03,0.12)^***^ | 0.87(0.83,0.92)^***^ | 90.89(36.68,225.23)^***^ | 1.15(1.09,1.21)^***^ | 0.41(0.15,1.12) | 1.02(0.99,1.05) |
| Other enteroviruses | 0.09(0.05,0.16)^***^ | 0.88(0.86,0.91)^***^ | 107.68(43.92,263.99)^***^ | 1.12(1.08,1.16)^***^ | 2.73(0.80,9.34) | 1.00(0.97,1.04) |
| Other enteroviruses (1-week lag) | 0.10(0.07,0.16)^***^ | 0.92(0.88,0.95)^***^ | 56.25(30.22,104.68)^***^ | 1.09(1.04,1.13)^***^ | 1.35(0.63,2.89) | 1.01(0.98,1.03) |
| Other enteroviruses (2-week lags) | 0.11(0.07,0.17)^***^ | 0.91(0.87,0.94)^***^ | 66.60(33.95,130.66)^***^ | 1.10(1.06,1.14)^***^ | 1.68(0.70,4.06) | 1.00(0.98,1.03) |
| IRR, incidence rate ratio. CI, confidence intervals. EV71, enterovirus 71. CV-A16, coxsackie virus A16. ^*^*P*<0.05 ^**^*P*<0.01 ^***^*P*<0.001 | | | | | | |

| Supplemental Table 2 Influence of different COVID control stages on hand, foot and mouth disease in the sensitivity analysis in Zhejiang, China | | | | | | |
| --- | --- | --- | --- | --- | --- | --- |
|  | Level change after strict control | Trend change after strict control | Level change after regular control^a^ | Trend change after regular control | Level change after reopening | Trend change after reopening |
|  | IRR（95%CI） | IRR（95%CI） | IRR（95%CI） | IRR（95%CI） | IRR（95%CI） | IRR（95%CI） |
| Overall | 0.26(0.15,0.47)^***^ | 0.75(0.66,0.86)^***^ | 24.87(5.45,113.48)^***^ | 1.33(1.17,1.51)^***^ | 1.26(0.30,5.30) | 1.01(0.97,1.06) |
| Sex |  |  |  |  |  |  |
| Male | 0.26(0.15,0.47)^***^ | 0.75(0.66,0.86)^***^ | 24.98(5.40,115.60)^***^ | 1.33(1.16,1.52)^***^ | 1.29(0.31,5.43) | 1.01(0.97,1.06) |
| Female | 0.26(0.15,0.47)^***^ | 0.75(0.67,0.86)^***^ | 24.77(5.52,111.20)^***^ | 1.33(1.17,1.50)^***^ | 1.22(0.29,5.11) | 1.01(0.97,1.06) |
| Age |  |  |  |  |  |  |
| <2 years | 0.33(0.17,0.62)^***^ | 0.75(0.66,0.86)^***^ | 19.21(4.08,90.50)^***^ | 1.32(1.16,1.51)^***^ | 1.80(0.40,8.12) | 1.01(0.96,1.06) |
| 2-4 years | 0.23(0.13,0.41)^***^ | 0.72(0.62,0.83)^***^ | 49.54(9.82,249.84)^***^ | 1.39(1.21,1.61)^***^ | 1.23(0.29,5.18) | 1.01(0.97,1.05) |
| ≥5 years | 0.25(0.14,0.44)^***^ | 0.83(0.75,0.92)^***^ | 8.45(2.28,31.40)^**^ | 1.21(1.09,1.34)^***^ | 0.77(0.19,3.16) | 1.02(0.98,1.07) |
| Groups |  |  |  |  |  |  |
| Children living separately | 0.29(0.16,0.54)^***^ | 0.76(0.66,0.87)^***^ | 20.98(4.35,101.25)^***^ | 1.32(1.15,1.52)^***^ | 1.58(0.35,7.17) | 1.01(0.96,1.06) |
| Children in kindergartens or nurseries | 0.24(0.14,0.42)^***^ | 0.66(0.57,0.76)^***^ | 114.01(22.34,581.71)^***^ | 1.53(1.32,1.76)^***^ | 1.02(0.25,4.20) | 1.01(0.97,1.05) |
| Students | 0.27(0.15,0.50)^***^ | 0.91(0.83,1.00)^*^ | 3.97(1.23,12.76)^*^ | 1.10(1.00,1.21)^*^ | 1.05(0.28,3.90) | 1.02(0.98,1.06) |
| Pathogens |  |  |  |  |  |  |
| EV71 | 0.77(0.44,1.35) | 0.67(0.59,0.76)^***^ | 22.39(5.89,85.07)^***^ | 1.51(1.32,1.72)^***^ | 0.53(0.13,2.09) | 1.05(1.01,1.08) |
| CV-A16 | 0.08(0.04,0.14)^***^ | 0.77(0.66,0.89)^***^ | 27.22(7.19,103.08)^***^ | 1.32(1.13,1.54)^***^ | 0.26(0.10,0.68)^**^ | 1.02(0.99,1.05) |
| Other enteroviruses | 0.13(0.07,0.21)^***^ | 0.77(0.71,0.84)^***^ | 68.44(22.22,210.79)^***^ | 1.29(1.18,1.41)^***^ | 2.49(0.72,8.66) | 1.00(0.96,1.04) |
| IRR, incidence rate ratio. CI, confidence intervals. EV71, enterovirus 71. CV-A16, coxsackie virus A16. ^*^*P*<0.05 ^**^*P*<0.01 ^***^*P*<0.001 ^a^From March 23^th^ 2020. | | | | | | |
